# Serum Levels of CXCL13 Are an Independent Predictor of Survival Following Resection of Biliary Tract Cancer

**DOI:** 10.3390/cancers14174073

**Published:** 2022-08-23

**Authors:** Sven H. Loosen, Tom F. Ulmer, Simon Labuhn, Jan Bednarsch, Sven A. Lang, Patrick H. Alizai, Anne T. Schneider, Mihael Vucur, Ulf P. Neumann, Tom Luedde, Christoph Roderburg

**Affiliations:** 1 Clinic for Gastroenterology, Hepatology and Infectious Diseases, University Hospital Düsseldorf, Medical Faculty of Heinrich Heine University Düsseldorf, 40225 Düsseldorf, Germany; 2 Department of Visceral and Transplantation Surgery, University Hospital RWTH Aachen, Pauwelsstrasse 30, 52074 Aachen, Germany

**Keywords:** chemokines, biomarker, biliary tract cancer, cholangiocarcinoma, chemokine (C-X-C motif) ligand, CXC chemokine receptors

## Abstract

**Simple Summary:**

Biliary tract cancer (BTC) is a primary liver malignancy with poor outcome. The identification of the ideal surgical candidates is often challenging and stratification algorithms comprising the parameters of individual tumor biology are missing. Here, we investigated a potential role of circulating CXCL1, CXCL10 and CXCL13 in patients with resectable BTC as novel biomarkers and could show that elevated levels of CXCL13 both before and after tumor resection identified a subgroup of patients with significantly impaired outcomes following tumor resection. Thus, the present study supports a fundamental role of the CXC chemokine family in BTC and identifies circulating levels of CXCL13 as a previously unrecognized marker for predicting outcomes following the resection of BTC.

**Abstract:**

Background: The prognosis of biliary tract cancer (BTC) has remained very poor. Although tumor resection represents a potentially curative therapy for selected patients, tumor recurrence is common, and 5-year survival rates have remained below 50%. As stratification algorithms comprising the parameters of individual tumor biology are missing, the identification of ideal patients for extensive tumor surgery is often challenging. The CXC chemokine family exerts decisive functions in cell–cell interactions and has only recently been associated with cancer, but little is known about their function in BTC. Here, we aim to evaluate a potential role of circulating CXCL1, CXCL10 and CXCL13 in patients with resectable BTC. Methods: Serum levels of CXCL1, CXCL10 and CXCL13 were measured by multiplex immunoassay in a cohort of 119 BTC patients undergoing tumor resection and 50 control samples. Results: Circulating levels of CXCL1, CXCL10 and CXCL13 were all significantly elevated in BTC patients compared to healthy controls and increased the diagnostic power of established tumor markers such as CA19-9 when used in combination. Importantly, elevated levels of CXCL13 both before and after tumor resection identified a subgroup of patients with significantly impaired outcomes following tumor resection. As such, BTC patients with initial CXCL13 levels above the ideal prognostic cut-off value (25.01 pg/mL) had a median overall survival (OS) of 290 days compared to 969 days for patients with low initial CXCL13 levels. The prognostic value of circulating CXCL13 was further confirmed by uni- and multivariate Cox regression analyses. Finally, the individual kinetics of CXCL13 before and after tumor resection were also indicative of patient outcomes. Conclusion: Our data support a fundamental role of the CXC chemokine family in BTC and identified circulating levels of CXCL13 as a previously unrecognized marker for predicting outcomes following the resection of BTC.

## 1. Introduction

Biliary tract cancer (BTC) represents a rare GI tumor entity with dismal outcomes [1]. Incidence rates have constantly risen during the last decades, but no specific therapeutic approaches for BTC have been established to date [1]. Tumor resection followed by adjuvant chemotherapy represents a potentially curative treatment option for selected BTC patients at early disease stages. However, larger clinical trials have shown that disease recurrence after tumor resection is observed in up to 60% of cases, leading to a 5-year survival rate of less than 50%, even for patients who received adjuvant chemotherapy [2]. Importantly, the decision for or against tumor resection is mostly based on surgical resectability and the patients’ performance status, while aspects of the individual BTC tumor biology are not considered [3]. In order to identify subgroups of patients that will particularly benefit from surgical tumor resection, novel easily accessible biomarkers are urgently needed to improve the outcomes for this highly aggressive GI malignancy.

BTC exhibits a complex microenvironment that is completely different from that of other primary liver cancers, such as HCC. The microenvironment of BTC is characterized by a dense tumor stroma and matricellular proteins, an abundance of cancer-associated fibroblasts and tumor-associated macrophages as well as innate immune cells [4,5]. Recent data highlighted a tremendous role of this complex microenvironment in promoting CCA progression, therapeutic resistance and immunosuppression [6,7,8,9]. Of note, it was not the tumor cells themselves but rather cancer-associated myofibroblasts (CAM) that were identified as the major source of the growth factors, cytokines and enzymes that drive BTC growth, tumor invasiveness and cell survival [5]. In line with these findings, CAM depletion demonstrated anti-tumor-progression effects in in vitro and in vivo assays of BTC [10].

C-X-C motif (CXC) chemokines represent a family of low-molecular-weight chemotactic cytokines with well-known functions in immune cell recruitment and cancer [11]. In particular, CXC ligand 1 (CXCL1), CXCL10 and CXCL13 have recently been associated with tumor survival, metastasis and angiogenesis [12,13]. As such, high tumoral CXCL1 expression was associated with increased tumor aggressiveness and poor outcomes in hepatocellular carcinoma (HCC) [12,13]. Moreover, CXCL10 and CXCL13 have both been linked to cancer progression and an unfavorable clinical course in colorectal carcinoma and HCC patients [14,15,16]. On the contrary, only very limited data on a potential function of these chemokines in the context of BTC exist [17].

Considering the lack of serum-based markers in the context of BTC, we evaluated circulating levels of CXCL1, CXCL10 and CXCL13 in a large cohort of BTC patients undergoing tumor resection between 2011 and 2017, with the aim to analyze their potential as diagnostic and prognostic markers in these patients.

## 2. Patients and Methods

### 2.1. Study Design and Patient Characteristics

This observational cohort study was conducted to evaluate a diagnostic and/or prognostic role of circulating CXC chemokines (CXCL1, CXCL10 and CXCL13) in patients with BTC undergoing tumor resection. A total of *n* = 119 BTC patients who were admitted to the Department of Visceral and Transplantation Surgery at University Hospital RWTH Aachen for tumor resection were recruited between 2011 and 2017 (see Table 1 and Table S1 for detailed patient characteristics). Blood samples were taken before surgery and 6–7 days after BTC resection and centrifuged for 10 min at 2000×  *g*, and serum samples were then stored at −80 °C until examination. BTC was confirmed histologically in the resected tumor samples. As a control population, we analyzed a total of *n* = 50 healthy cancer-free blood donors. Overall survival was defined as the time period between surgical resection and death. The date of death was obtained either from internal documents of our clinic, after consultation with the patient’s general practitioners, or by querying the death register of the registry office. Patients for whom no proof of death was available at the time of analysis were censored at the time of the last physician contact (clinic or general practitioner). The study protocol was approved by the ethics committee of the University Hospital RWTH Aachen, Germany (EK 206/09) and conducted in accordance with the ethical standards laid down in the Declaration of Helsinki. Written informed consent was obtained from all patients.

### 2.2. Evaluation of Circulating CXC Chemokine Levels

Serum levels of CXCL1, CXCL10 and CXCL13 were analyzed by multiplex immunoassay, according to the manufacturer’s instruction, using a Bio-Plex 200 system and Bio-Plex Manager 6.0 software (Bio-Plex Pro Human Chemokine Panel, #171AK99MR2, Bio Rad, Hercules, CA, USA). A serum sample volume of 50 µL was used. The assay’s working ranges are 3.1–7024 pg/mL (CXCL1), 1.6–7714 pg/mL (CXCL10) and 0.7–1200 pg/mL (CXCL13).

### 2.3. Statistical Analysis

Statistical analyses were performed as recently described [18]. In brief, a Shapiro–Wilk test was used to test for a normal distribution. Nonparametric data were compared using the Mann–Whitney U test or the Kruskal–Wallis test for multiple group comparisons. Related samples were compared using the Wilcoxon signed-rank test. Correlation analyses were performed using Spearman’s correlation coefficient. Optimal cut-off values for the ROC curves were calculated with the Youden index method (YI = sensitivity + specificity − 1). Kaplan–Meier curves display the impact of a specific parameter on the overall survival (OS). A log-rank test was performed to test for differences between groups. The optimal prognostic cut-off value was determined by fitting Cox proportional hazard models to the dichotomized survival status and the survival time and defining the optimal cut-off as the point with the most significant split in the log-rank test. The prognostic relevance of variables was also tested in uni- and multivariate Cox regression analyses. Parameters with a *p*-value of <0.200 in univariate testing were included in multivariate testing. The hazard ratio (HR) and the 95% confidence interval are displayed. All statistical analyses were performed with SPSS 23 (SPSS, Chicago, IL, USA) and RStudio 1.2.5033 (RStudio Inc., Boston, MA, USA) [19]. A *p*-value of <0.05 was considered statistically significant (* *p* < 0.05; ** *p* < 0.01; *** *p* < 0.001). In the case of multiple comparisons (correlation analyses), the significance level was adjusted for multiple comparisons according to the Bonferroni correction method. A *p*-value of *p* = 0.004 was considered statistically significant.

## 3. Results

### 3.1. Serum Levels of Circulating CXC Chemokines Are Elevated in Biliary Tract Cancer Patients

**Figure 1:**
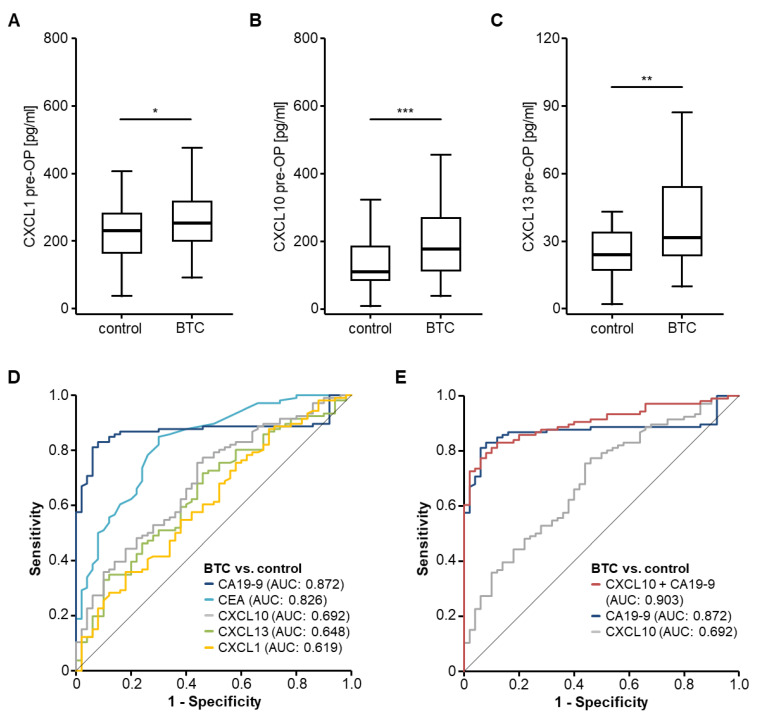
Circulating chemokine levels in biliary tract cancer patients. (**A**–**C**) BTC patients have significantly elevated serum levels of CXCL1, CXCL10 and CXCL13 compared to healthy controls. (**D**) Circulating chemokine levels show lower AUC values for the discrimination between BTC patients and healthy controls compared to established tumor markers such as CEA and CA19-9. (**E**) The combination of CXCL10 and CA19-9 further increases the AUC value for the identification of BTC cancer patients. * *p* < 0.05; ** *p* < 0.01; *** *p* < 0.001.

Based on available data on the dysregulation of several circulating CXC chemokines in HCC [15,16], we first hypothesized that serum levels of CXCL1, CXCL10 and CXCL13 might also be altered in patients with BTC. We therefore compared circulating chemokine levels in our cohort of *n* = 119 BTC patients with *n* = 50 healthy control samples. Interestingly, we found significantly elevated serum levels of all three chemokines in BTC patients (Figure 1A–C). While CXCL1 levels only showed a mild 1.1-fold induction (Figure 1A), both CXCL10 (1.6-fold, Figure 1B) and CXCL13 (1.3-fold, Figure 1C) serum levels were elevated to a higher extent in BTC patients compared to healthy controls. An ROC curve analysis revealed AUC values of AUC_CXCL1_: 0.619, AUC_CXCL10_: 0.692 and AUC_CXCL13_: 0.648 for the identification of BTC patients (Figure 1D). However, these AUC values were lower than those of established BTC tumor markers such as CEA (AUC_CEA_: 0.826) or CA19-9 (AUC_CA19-9_: 0.872). Nevertheless, the combination of CXC chemokines with known tumor markers (e.g., CXCL10 and CA19-9) increased the diagnostic potential compared to either parameter alone, showing an AUC value of 0.903 (Figure 1E). The sensitivity and specificity of the combined panel (CA19-9/CXCL10) were 77.4% and 94.0%, respectively. The AUC of other combinations with existing tumor markers are displayed in Table S3.

### 3.2. CXC Chemokine Serum Levels and Patient Characteristics

We next compared the circulating levels of CXCL1, CXCL10 and CXCL13 between BTC patients with different tumor and clinical characteristics. The baseline serum levels of CXCL1 were comparable between patients with different tumor localizations (iCCA, Klatskin, distal CCA and gallbladder cancer), T stage, M stage (who were still eligible for tumor resection), tumor grading or resection status (Figure S1A,B,D–F), while patients with node-positive disease (N1) showed slightly lower CXCL1 serum levels (Figure S1C). Moreover, the CXCL1 serum levels did not differ between male and female patients (Figure S1G) and patients of different ECOG performance status (Figure S1H). The initial serum levels of CXCL10 were significantly higher in patients with distal CCA compared to other tumor localizations (Figure S2A) but were unaltered between patients with different TNM stages, tumor grading or resection status (Figure S2B–F). While CXCL10 serum levels were comparable between male and female patients, we observed significantly higher levels in patients with an impaired ECOG PS (Figure S2G,H). Finally, baseline levels of CXCL13 were unaltered between patients with different tumor localizations, TNM stages, tumor grading, resection status or sex but were significantly higher in patients with an impaired ECOG performance status (Figure S3A–H). In correlation analyses including laboratory parameters of organ dysfunction as well as clinical parameters, there were no significant correlations between patients’ age or BMI and preoperative CXCL1, CXCL10 or CXCL13 levels (Table S2). However, we observed a significant correlation between preoperative CXCL10 levels and laboratory parameters of liver dysfunction (AST: R_s_ = 0.304, *p* = 0.001; bilirubin: R_s_ = 0.370, *p* = 0.001; ALP: R_s_ = 0.286, *p* = 0.002) as well as between baseline CXCL13 levels and CRP (R_s_ = 0.344, *p* = 0.001, Table S2).

### 3.3. Baseline CXCL13 Levels Are an Independent Predictor of Postoperative Survival following BTC Resection

**Figure 2:**
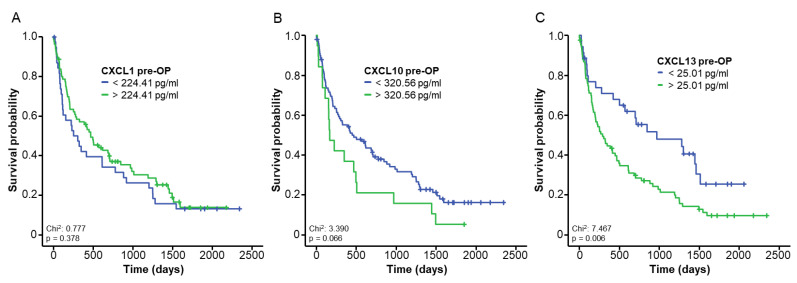
Elevated baseline levels of CXCL13 indicate poor overall survival after BTC resection. (**A**–**C**) Overall survival in BTC patients with initial CXCL1, CXCL10 and CXCL13 levels above or below the ideal prognostic cut-off value.

We next hypothesized that the elevation in CXCL1, CXCL10 and CXCL13 in BTC patients might have a prognostic relevance following tumor resection. We therefore split our cohort into two groups based on the median serum level of the respective chemokine (CXCL1: 253.44 pg/mL, CXCL10: 177.02 pg/mL and CXCL13: 31.68 pg/mL) and compared the overall survival (OS) of these groups using Kaplan–Meier estimates. The median OS was comparable between the CXCL1 (*p* = 0.846), CXCL10 (*p* = 0.357) and CXCL13 (*p* = 0.110) high/low groups. In a next step, we determined optimal prognostic cut-off values (see Patients and Methods for details) that best discriminated between patients with good or poor postoperative outcomes (CXCL1: 224.41 pg/mL, CXCL10: 320.56 pg/mL and CXCL13: 25.01 pg/mL). After applying these ideal cut-off values, CXCL1 and CXCL10 serum levels were still unsuitable to predict postoperative outcomes (Figure 2A,B). Importantly, we observed a significantly reduced OS in patients with initial CXCL13 serum levels above the ideal cut-off value (25.01 pg/mL) compared to patients with low baseline CXCL13 levels (Figure 2C). The median OS of BTC patients with CXCL13 levels above 25.01 pg/mL was only 290 days compared to 969 days for patients with CXCL13 levels below 25.01 pg/mL (Figure 2C).

To further corroborate the prognostic relevance of CXCL13 and to identify potential confounders, we next performed uni- and multivariate Cox regression analyses including several clinicopathological parameters of potential prognostic relevance. In univariate testing, initial CXCL13 serum levels above 25.01 pg/mL were a significant prognostic factor for OS (HR: 1.993, 95% CI: 1.195–3.128, *p* = 0.007). Moreover, the univariate analyses revealed prognostic relevance (*p* < 0.200) for CEA, CRP and creatinine serum levels and hemoglobin levels as well as the ECOG PS, age and the tumor stage (Table 2). Importantly, when including these parameters in the multivariate analysis, the serum CXCL13 levels stood out as an independent prognostic parameter for OS (HR: 2.094, 95% CI: 1.020–4.297, *p* = 0.044, Table 2).

### 3.4. Postoperative Levels of CXCL10 and CXCL13 Predict Outcome following BTC Resection

**Figure 3:**
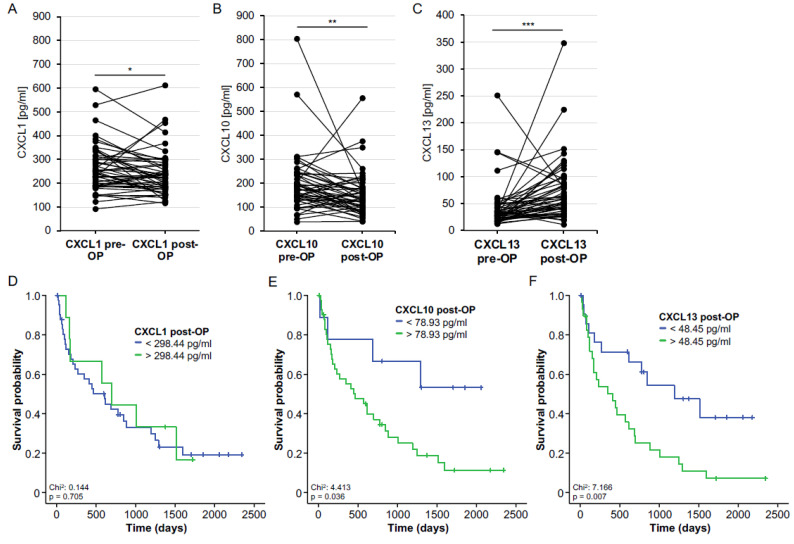
Postoperative chemokine levels and outcomes. (**A**) Serum levels of CXCL1 were significantly lower after BTC resection. (**B**) Serum levels of CXCL10 were significantly lower after BTC resection. (**C**) Serum levels of CXCL13 were significantly higher after BTC resection. (**D**–**F**) Overall survival in BTC patients with postoperative CXCL1, CXCL10 and CXCL13 levels above or below the ideal prognostic cut-off values. * *p* < 0.05; ** *p* < 0.01; *** *p* < 0.001.

Based on the promising findings on the prognostic relevance of baseline CXCL13 serum levels, we subsequently evaluated the potential regulation of CXCL1, CXCL10 and CXCL13 after tumor resection. Postoperative serum samples were available for *n* = 52 BTC patients. While CXCL1 and CXCL10 levels significantly decreased after tumor resection (Figure 3A,B), postoperative CXCL13 levels were significantly higher compared to preoperative values (Figure 3C). We again established ideal prognostic cut-off values to evaluate a potential prognostic function of postoperative chemokine levels (CXCL1: 298.44 pg/mL, CXCL10: 78.93 pg/mL and CXCL13: 48.45 pg/mL). Although the Kaplan–Meier curve analyses revealed no prognostic relevance of postoperative CXCL1 levels (Figure 3D), we observed significantly reduced OS in patients with either CXCL10 or CXL13 levels above the ideal postoperative cut-off values (78.93 pg/mL and 48.45 pg/mL) compared to patients with chemokine levels below these cut-offs (Figure 3E,F). In line with these results, Cox regression analyses revealed both postoperative CXCL10 and CXCL13 serum levels as prognostic factors for OS (HR_CXCL10_: 2.948, 95% CI: 1.036–8.391, *p* = 0.043; HR_CXCL13_: 2.518, 95% CI: 1.242–5.106, *p* = 0.010).

**Figure 4:**
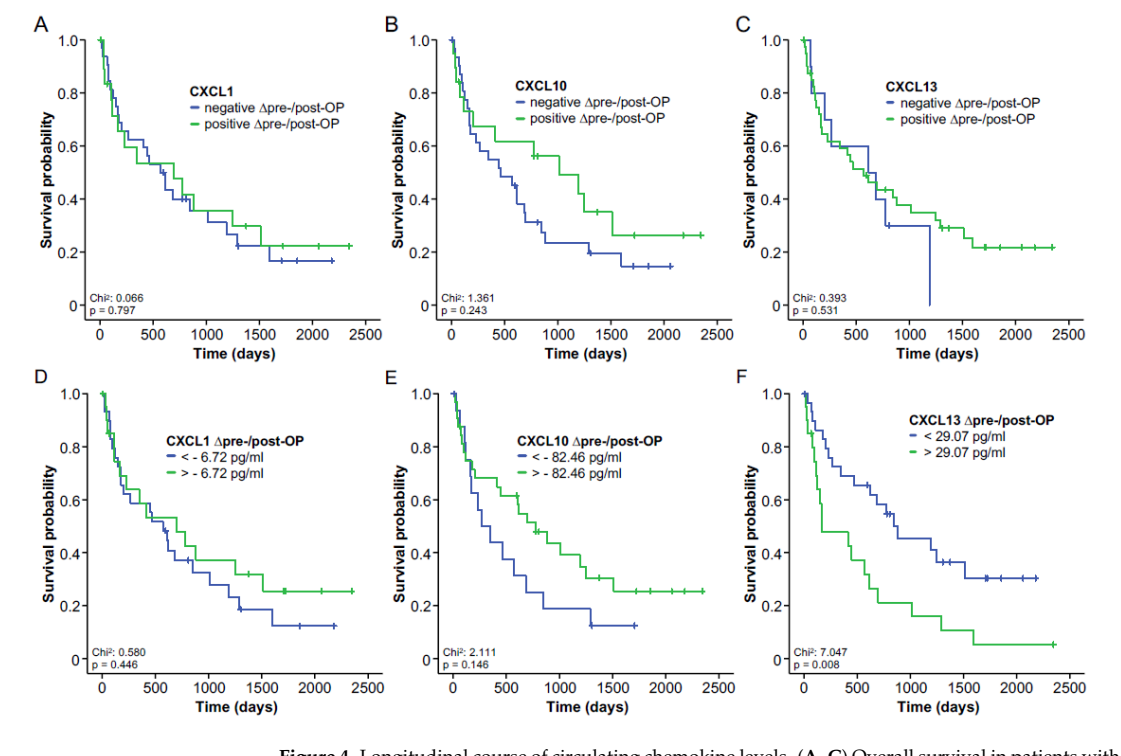
Longitudinal course of circulating chemokine levels. (**A**–**C**) Overall survival in patients with increasing or decreasing CXCL1, CXCL10 and CXCL13 levels. (**D**–**F**) Overall survival in patients with increasing or decreasing CXCL1, CXCL10 and CXCL13 levels using ideal prognostic cut-off values.

In a next step, we aimed to evaluate whether the individual kinetics of circulating chemokine levels before and after tumor resection might also reflect postoperative outcomes. We therefore compared the OS of patients with increasing or decreasing chemokine levels using Kaplan–Meier estimates. However, we did not observe a significant survival benefit for either group with respect to CXCL1, CXCL10 or CXCL13 serum levels (Figure 4A–C). We then further dissected the individual kinetics of circulating chemokine levels before and after tumor resection and identified ideal cut-off values at which the prognostic potential was highest. Here, the Kaplan–Meier curve analysis revealed significantly impaired OS in BTC patients who showed strongly increasing CXCL13 levels (>29.07 pg/mL) after tumor resection compared to patients with decreasing or only mildly increasing CXCL13 levels (Figure 4F). In contrast, the individual kinetics of CXCL1 and CXCL10 levels were unsuitable to predict postoperative outcomes (Figure 4D,E).

Finally, we intended to gain further insights into the underlying mechanisms associated with the postoperative downregulation of CXCL1 and CXL10 and the upregulation of CXCL13. We therefore performed extensive correlation analyses between the postoperative levels of CXCL1, CXCL10 and CXCL13 and various markers of organ dysfunction as well as clinical parameters. Here, we observed a positive correlation between postoperative CXCL13 levels and the leucocyte count (R_S_: 0.317, *p* = 0.025), suggesting that postoperative systemic inflammation was a potential driver of elevated CXCL13 levels after tumor resection (Table S2). There was no correlation between chemokine levels and markers of impaired liver or renal function or patients’ age and BMI (Table S2).

## 4. Discussion

Biliary tract cancer (BTC) represents a group of aggressive GI malignancies with poor outcomes [1]. Although tumor resection can potentially provide a cure for selected patients, up to 50% of patients face disease recurrence limiting their overall prognosis decisively [20]. While several predictive factors for tumor recurrence and outcome have been suggested [21], the reliable identification of ideal surgical candidates has remained a major challenge. In this context, it is important to note that the decision for or against tumor resection is primarily based on the patients’ clinical status and imaging techniques providing information on the surgical resectability, while aspects of the tumor biology are basically not considered. In this study, we provide evidence that circulating levels of CXCL1, CXCL10 and CXCL13 are distinctly altered in patients with BTC and that serum CXCL13 levels, in particular, represent a promising candidate to predict outcomes following tumor resection. As an example, initial CXCL13 levels above the ideal prognostic cut-off value that we established in our study (25.01 pg/mL) identified a subgroup of BTC patients with significantly impaired median OS compared to the subgroup of patients with baseline CXCL13 levels below this cut-off (290 vs. 969 days). Multivariate Cox regression analyses including several clinicopathological parameters confirmed this finding. Similarly, postoperative CXCL13 as well as CXCL10 levels significantly predicted outcomes after BTC resection.

In addition to their well-established function in inflammatory processes and immune responses, CXCL chemokines and their respective receptors (CXCR) exert important molecular functions in the context of cancer. Particularly, the signaling of CXCL13 through its receptor, CXCR5, has recently been associated with promalignant intracellular pathways such as PI3K/AKT, MEK/ERK and Rac-GEF/Rac, leading to cell survival, proliferation and migration in different tumor entities [22]. In the field of GI cancer, a promalignant role of the CXCL13/CXCR5 axis has been shown for pancreatic cancer and gastric cancer as well as hepatocellular carcinoma [15,23]. In pancreatic cancer, which shares many similarities with BTC, high expression of CXCR5 was observed in human pancreatic cancer cell lines as well as human tissue samples [24]. Moreover, these cell lines display a strong elevation in noncanonical NF-kB target genes, including CXCL13 [25]. A constitutive activation of the noncanonical NF-kB pathway, which was observed in different models of pancreatic cancer, might thus result in a autocrine promigratory cytokine loop through the activation of CXCR5 [22,25]. In addition, CXCL13 has been suggested as a crucial pancreatic B-cell chemoattractant associated with proneoplastic functions in a Kras-driven pancreatic cancer model [26]. Elevated CXCL13 and CXCR5 expression has also been described in hepatocellular carcinoma (HCC) and was suggested to promote cancer growth through the Wnt/ß-catenin pathway [27,28]. Interestingly, tumoral overexpression was reflected by elevated serum levels in HCC patients and correlated with disease stage and recurrence-free survival [27,28]. In the context of BTC, however, no data on a potential role of circulating CXCL13 levels exist. Although our study does not provide information on a functional role of CXCL13, linking elevated CXCL13 serum levels with impaired outcomes following the resection of BTC, our data suggest that high CXCL13 serum levels might reflect a more aggressive cancer. Importantly, this hypothesis clearly needs to be addressed in future molecular studies further dissecting a potential functional role of CXCL13 in BTC, ideally by using CXCL13- or CXCR5-deficient mice [29]. Similarly, our data on elevated levels of CXCL10 and CXCL1 in the serum of patients with BTC are interesting. Recently, CXCL10 was identified as part of a six-chemokine signature comprising CCL20, C-reactive protein, CXCL8, CXCL10, resistin and serum amyloid A [30]. Moreover, Yamamoto et al. recently suggested an important role of CXCL1-CXCR2 signaling in BTC [31], highlighting that biologically plausible sets of cytokines/chemokines might play roles as diagnostic markers in patients with BTC.

In a subgroup of patients, we analyzed circulating chemokine levels 6 to 7 days after tumor resection to gain further insight into the longitudinal regulation of these markers. Interestingly, while the serum levels of CXCL1 and CXCL10 decreased postoperatively, we observed significantly increased values of CXCL13 after tumor removal. This fact argues against the hypothesis that circulating CXCL13 exclusively originated from BTC tumor cells, suggesting that they reflect a status of systemic inflammation that might originate from the BTC as well as the tumor microenvironment [32]. In our study, postoperative systemic inflammation, which is regularly observed following major abdominal surgery, represents the most likely cause of increasing postoperative CXCL13 levels. Along this line of thinking, we observed a positive correlation between postoperative CXCL13 levels and the leucocyte count, which is in good agreement with data on elevated CXCL13 levels in patients with systemic inflammation or sepsis [33]. Together, these data suggest that future studies should also concentrate on later time-points after tumor resection to fully illuminate the kinetics of circulating CXCL13 after tumor resection.

A potential clinical implication of circulating CXCL13 as a novel biomarker for patient stratification before tumor resection should be considered with caution. It is rather unlikely that physicians and/or BTC patients would decide against a potentially curative tumor resection if it were technically feasible based on a preoperative CXCL13 elevation above the prognostic cut-off value. We suggest that circulating CXCL13 levels should be implemented into existing stratification tools, rather than being used as a stand-alone parameter. As more aggressive adjuvant chemotherapy regimens are under clinical evaluation, elevated CXCL13 levels before and/or after BTC resection could also identify a subgroup of patients that should receive highly active postoperative therapy to reduce the likelihood of disease recurrence and improve their outcomes. Along this line of thinking, the evaluation of CXCL13 levels in previous and ongoing larger clinical trials on resectable BTC might provide further evidence for this hypothesis [2].

The quality of our study was limited by some aspects. First of all, our study concentrated on surgical tumor resection only and did not include alternative treatment approaches, such as systemic chemotherapy. Along this line of thinking, we cannot provide information regarding the question of whether an individual patient with high baseline CXCL13 levels might have benefitted to the same or even a higher extent from nonsurgical therapy. Second, the study was conducted in a single-center design. Although, this fact might prevent interhospital bias, larger multicenter approaches are needed to confirm our findings. Third, the healthy control cohort of blood donors was not matched for patient age. Fourth, the prognostic analyses focused on OS only and did not include other relevant endpoints, such as the progression-free survival or the occurrence of complications. Finally, our study does not allow any conclusions on a functional role of CXCL13 in the context of BTC. Along this line of thinking, the immunohistochemical evaluation of selected chemokine receptors in BTC tumor tissue samples, which were not feasible in the current study, could yield important information in the future.

## 5. Conclusions

Together, our study suggests a potential role of CXCL13 levels as a previously unrecognized easily accessible biomarker in the context of BTC. While our data need further validation from larger and independent BTC cohorts, they argue that CXCL13 levels might support the decision making of clinicians in terms of surgical tumor resection.

## Figures and Tables

**Table 1:** Patient characteristics.

	BTC Patients	Healthy Controls	*p*-Value
Number of individuals (total)	119	50	
Gender (%): male-female	55.1-44.9	72.9-27.1	0.054
Age (years, median and range)	68 [37–84]	37 [19–74]	<0.001
BMI (kg/m^2^, median and range)	25.75 [18.83–46.36]	24.64 [18.94–56.01]	0.139
Anatomic location of BTC (%)			
Intrahepatic	42.0	-	
Klatskin	40.3	-	
Distal	10.1	-	
Gallbladder	7.6	-	
Staging (%)			
T1–T2–T3–T4	11.0–35.0–36.0–18.0	-	
N0–N1	46.2–53.8	-	
M0–M1	82.5–17.5	-	
G2–G3	59.3–40.7	-	
R0–R1	65.6–34.4	-	
ECOG PS (%)			
ECOG 0	50.5	-	
ECOG 1	40.2	-	
ECOG 2	9.3	-	

BTC: biliary tract cancer, BMI: body mass index, ECOG PS: “Eastern Cooperative Oncology Group” performance status.

**Table 2:** Uni- and multivariate Cox regression analyses of preoperative parameters for the prediction of overall survival.

	Univariate Cox Regression		Multivariate Cox Regression	
Parameter	*p*-Value	Hazard Ratio (95% CI)	*p*-Value	Hazard Ratio (95% CI)
CXCL13 > 25.01	0.007	1.993 (1.195–3.128)	0.044	2.094 (1.020–4.297)
CA19-9	0.290	1.000 (1.000–1.000)		
CEA	0.041	1.004 (1.000–1.009)	0.461	1.002 (0.997–1.007)
Leukocytes	0.542	1.020 (0.958–1.085)		
CRP	<0.001	1.008 (1.004–1.012)	0.284	1.005 (0.996–1.014)
Platelets	0.825	1.000 (0.999–1.002)		
Hemoglobin	0.012	0.817 (0.699–0.956)	0.111	0.800 (0.608–1.053)
Potassium	0.251	1.282 (0.839–1.960)		
AST	0.421	0.999 (0.998–1.001)		
Bilirubin	0.640	0.985 (0.927–1.048)		
ALP	0.375	1.000 (0.999–1.002)		
GGT	0.977	1.000 (1.000–1.000)		
Creatinine	0.020	2.437 (1.147–5.175)	0.581	0.742 (0.257–2.144)
BMI	0.591	1.011 (0.971–1.053)		
ECOG PS	0.119	1.329 (0.929–1.901)	0.963	1.012 (0.619–1.653)
Age	0.037	1.021 (1.001–1.042)	0.776	1.004 (0.974–1.035)
Sex	0.773	0.941 (0.624–1.420)		
T stage	0.004	1.530 (1.149–2.036)	0.250	1.256 (0.852–1.853)

CI: confidence interval, CXCL: chemokine (C-X-C motif) ligand, CEA: carcinoembryogenic antigen, CA19-9: carbohydrate antigen 19-9, CRP: C-reactive protein, AST: aspartate transaminase, ALP: alkaline phosphatase, GGT: Gamma-glutamyltransferase, BMI: body mass index, ECOG PS: “Eastern Cooperative Oncology Group” performance status.

## Data Availability

The data are available upon reasonable request from the corresponding author.

## Supplementary Materials

The following supporting information can be downloaded at: https://www.mdpi.com/article/10.3390/cancers14174073/s1, Figure S1: Baseline serum levels of CXCL1 are comparable between patients with different tumor localizations; Figure S2. Initial serum levels of CXCL10 are significantly higher in patients with distal CCA, compared to other tumor localizations; Figure S3. Baseline levels of CXCL13 are unaltered between patients with different tumor localizations; Table S1. Serum concentrations of chemokines and other laboratory parameters.; Table S2. Correlation analysis between postoperative CXCL1, CXCL10, and CXCL13 levels and markers of organ dysfunction.
