# Supplementary material for: Serum Levels of CXCL13 Are an Independent Predictor of Survival Following Resection of Biliary Tract Cancer
Source: Cancers (Basel). 2022 Aug 23;14(17):4073. doi: 10.3390/cancers14174073 (PMC9454558; doi:10.3390/cancers14174073)
Supplement: Supplementary file 1 [file cancers-14-04073-s001.zip › cancers-1838077-supplementary.pdf]

cancers

MDPI

Article

Serum levels of CXCL13 Are an Independent Predictor of Survival Following Resection of Biliary Tract Cancer

Sven H. Loosen [1], Tom F. Ulmer [2], Simon Labuhn [1], Jan Bednarsch [2], Sven A. Lang [2], Patrick H. Alizai [2], Anne T. Schneider [1], Mihael Vucur [1], Ulf P. Neumann [2], Tom Luedde [1,†] and Christoph Roderburg [1,†]

Table S1. Serum concentrations of chemokines and other laboratory parameters.

Laboratory parameter	BTC patients (n=119)	Healthy controls (n=50)
CXCL1 pre-OP [pg/ml]	253.44 [91.64-999.30]	231.24 [38.64-1613.34]
CXCL10 pre-OP [pg/ml]	178.44 [38.37-5211.22]	109.88 [7.83-419.68]
CXCL13 pre-OP [pg/ml]	32.01 [10.02-343.09]	24.07 [2.18-158.01]
CXCL1 post-OP [pg/ml]	223.84 [115.49-611.17]	-
CXCL10 post-OP [pg/ml]	133.16 [40.57-566.69]	-
CXCL13 post-OP [pg/ml]	58.33 [10.92-348.01]	-
CEA [µg/l]	2.95 [0.71-333.0]	1.25 [0.30-6.30]
CA 19-9 [U/ml]	74.30 [0.60-38092.0]	5.40 [0.0-44.10]
Leukocyte count [cells/nl]	7.90 [2.90-21.60]	
CRP [mg/l]	17.0 [0.0-230.0]	
Platelets [cells/nl]	263.50 [65.0-931]	
Hemoglobin [g/l]	12.50 [7.80-17.10]	
Sodium [mmol/l]	139.50 [131.0-146.0]	
Potassium [mmol/l]	4.30 [2.90-6.10]	
AST [U/l]	45.0 [17.0-1587.0]	28.0 [20.0-78.0]
Bilirubin [mg/dl]	0.93 [0.24-21.49]	0.41 [0.10-1.46]
ALP [U/l]	202.0 [52.0-1055.0]	65.0 [36.0-102.0]
Creatinine [mg/dl]	0.90 [0.42-2.08]	

BTC: biliary tract cancer, CXCL: chemokine (C-X-C motif) ligand, CEA: carcinoembryonic antigen, CA 19-9: carbohydrate-Antigen 19-9, CRP: C-reactive protein, AST: aspartate transaminase, ALP: alkaline phosphatase.

Table S2. Correlation analysis between postoperative CXCL1, CXCL10, and CXCL13 levels and markers of organ dysfunction.

Parameter	CXCL10		CXCL10		CXCL13	
	R_S	p-value	R_S	p-value	R_S	p-value
Leukocyte count	0.064	0.657	0.085	0.557	0.317	0.025
CRP	0.116	0.436	0.085	0.568	0.286	0.051
Platelets	0.249	0.081	0.262	0.066	0.126	0.384
Hemoglobin	-0.239	0.095	0.144	0.318	-0.056	0.699
Sodium	0.113	0.437	-0.154	0.285	-0.064	0.658
Potassium	0.012	0.936	0.025	0.863	0.017	0.909
AST	-0.094	0.515	0.076	0.602	-0.107	0.459
Bilirubin	0.158	0.280	0.217	0.135	-0.127	0.383
ALP	0.212	0.157	0.257	0.085	-0.117	0.440
Creatinine	0.039	0.787	0.150	0.299	0.139	0.336

CXCL: chemokine (C-X-C motif) ligand, CRP: C-reactive protein, AST: aspartate transaminase, ALP: alkaline phosphatase.

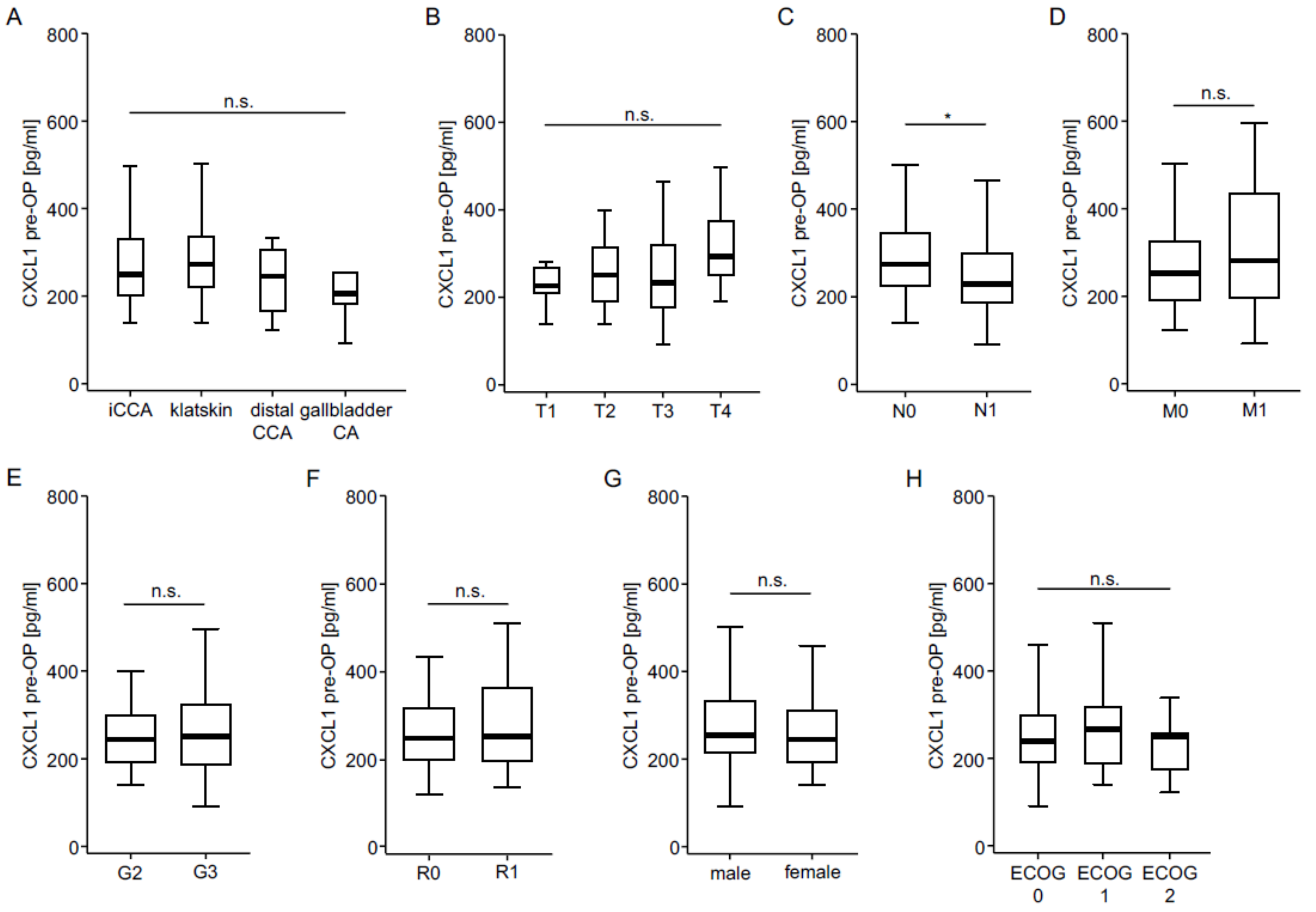

Figure S1. Baseline serum levels of CXCL1 are comparable between patients with different tumor localizations (A), T-stage (B), M-stage (D), tumor grading (E) or resection status (F), while patients with nodal positive disease (N1) show slightly lower CXCL1 serum levels (C). Moreover, CXCL1 serum levels DO not differ between male and female patients (G) and patients with different ECOG performance status (H).

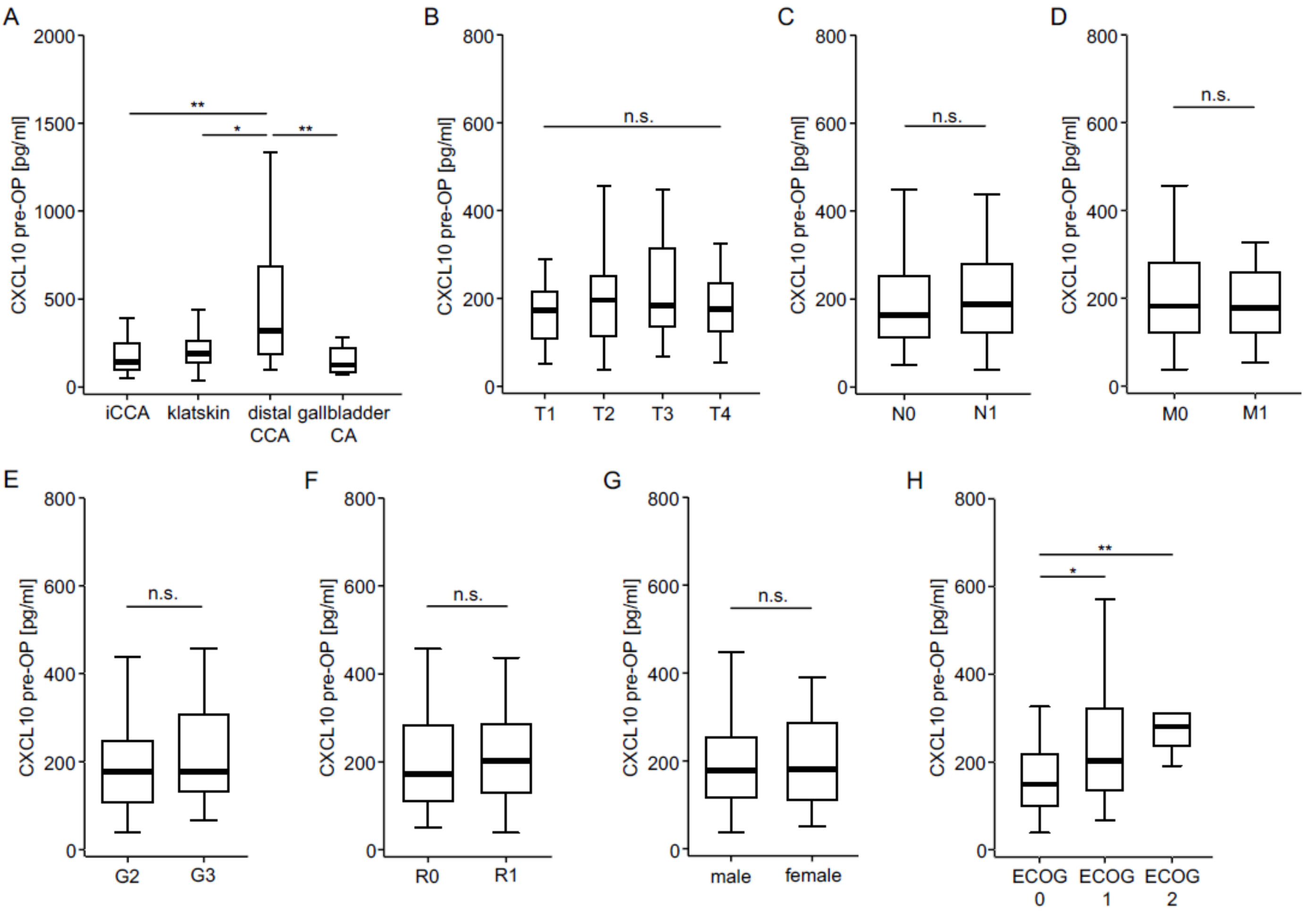

Figure S2. Initial serum levels of CXCL10 are significantly higher in patients with distal CCA, compared to other tumor localizations (A), but unaltered between patients with different TNM-stage (B-D), tumor grading (E) or resection status (F). While CXCL10 serum levels are comparable between male and female patients (G), patients with an impaired ECOG PS have significantly higher serum levels (H).

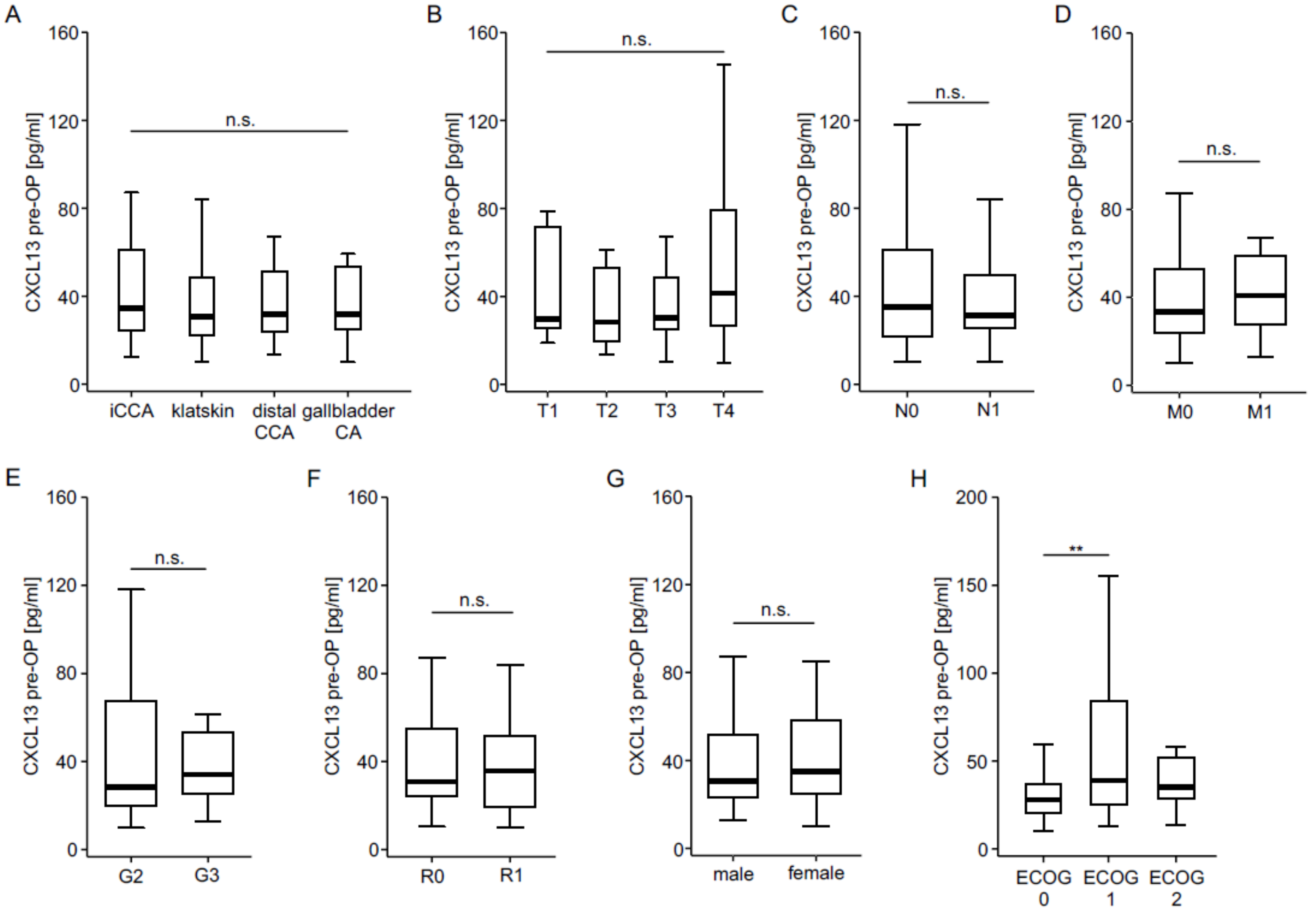

Figure S3. Baseline levels of CXCL13 are unaltered between patients with different tumor localizations (A), TNM-stage (B-D), tumor grading (E), resection status (F) or sex (G), but significantly higher in patients with an impaired performance status (H).
